# A 3-Dimensional Trimeric β-Barrel Model for *Chlamydia* MOMP Contains Conserved and Novel Elements of Gram-Negative Bacterial Porins

**DOI:** 10.1371/journal.pone.0068934

**Published:** 2013-07-25

**Authors:** Victoria A. Feher, Arlo Randall, Pierre Baldi, Robin M. Bush, Luis M. de la Maza, Rommie E. Amaro

**Affiliations:** 1 Department Chemistry and Biochemistry, University of California San Diego, San Diego, California, United States of America; 2 School of Information and Computer Sciences, University of California Irvine, Irvine, California, United States of America; 3 Institute for Genomics and Bioinformatics, University of California Irvine, Irvine, California, United States of America; 4 Department of Ecology and Evolutionary Biology, University of California Irvine, Irvine, California, United States of America; 5 Department of Pathology and Laboratory Medicine, University of California Irvine, Irvine, California, United States of America; National Research Council of Italy, Italy

## Abstract

*Chlamydia trachomatis* is the most prevalent cause of bacterial sexually transmitted diseases and the leading cause of preventable blindness worldwide. Global control of *Chlamydia* will best be achieved with a vaccine, a primary target for which is the major outer membrane protein, MOMP, which comprises ∼60% of the outer membrane protein mass of this bacterium. In the absence of experimental structural information on MOMP, three previously published topology models presumed a16-stranded barrel architecture. Here, we use the latest β-barrel prediction algorithms, previous 2D topology modeling results, and comparative modeling methodology to build a 3D model based on the 16-stranded, trimeric assumption. We find that while a 3D MOMP model captures many structural hallmarks of a trimeric 16-stranded β-barrel porin, and is consistent with most of the experimental evidence for MOMP, MOMP residues 320–334 cannot be modeled as β-strands that span the entire membrane, as is consistently observed in published 16-stranded β-barrel crystal structures. Given the ambiguous results for β-strand delineation found in this study, recent publications of membrane β-barrel structures breaking with the canonical rule for an even number of β-strands, findings of β-barrels with strand-exchanged oligomeric conformations, and alternate folds dependent upon the lifecycle of the bacterium, we suggest that although the MOMP porin structure incorporates canonical 16-stranded conformations, it may have novel oligomeric or dynamic structural changes accounting for the discrepancies observed.

## Introduction

*C. trachomatis*, a gram-negative bacterium, is estimated to infect 90 million people worldwide each year [1]. In some individuals these infections cause long-term consequences including pelvic inflammatory disease, ectopic pregnancy, sterility or blindness [2]–[5]. Although *Chlamydia* can be treated with anti-bacterials, many infections are asymptomatic and therefore go untreated [3], [6]. A prophylactic vaccine has long been sought against *C. trachomatis* but has yet to be obtained [7]–[9].

The *Chlamydia* major outer membrane protein, MOMP, is a primary target of vaccine development because it is highly antigenic and comprises ∼60% of the outer membrane protein mass [8], [10]. MOMP, coded by the *ompA* gene, is considered a member of the general porin class of proteins (http://scop.mrc-lmb.cam.ac.uk/scop) [11], a group important for the passive transport of ions, sugars and nucleotides across the outer membrane of gram-negative bacteria [12]–[14]. Porins have a structural topology comprised of antiparallel β-strands spanning the outer membrane, a water-filled inner channel, tight β-turns extending into the periplasmic region and flexible loops reaching beyond the extracellular surface. Several studies have confirmed that the *C. trachomatis* MOMP shares the characteristics of other gram-negative bacterial porins; it has a monomeric molecular weight of 39.5 kDa, a hydrophobic residue content of ∼40%, and migration patterns on SDS-page gels consistent with trimeric oligomerization [15]. Native MOMP also transports sugars at rates similar to *Pseudomonas aeruginosa* OprF [15]–[17].

Structural characterization of MOMP remains elusive for a number of reasons. Attempts to generate crystals for X-ray crystallographic studies with native MOMP proteins have been unsuccessful. Recombinant *Chlamydia* MOMP protein has been expressed in other systems but has been intractable due to inclusion body formation and very low refolding yields, most likely due to its highly hydrophobic nature and presence of a high number of cysteine residues. Notably, *Chlamydia* does not have an *available genetic system like other bacteria, such as E. coli, and only recently a transformation system limited to the plasmid was described* [18]. Yet, there is an urgent need to elucidate MOMP structural details and understand the conformation of its protective epitopes in order to formulate a vaccine.

Computational homology modeling is a useful alternative method for generating protein structural models in the absence of experimental structural data. However, direct homology modeling methods cannot be performed for the MOMP because it has little detectable sequence identity to β-barrels with solved structures. In general, protein structure is much more conserved than sequence as species diverge, often to the point where no detectable sequence similarity exists between pairs of sequences sharing the same structural fold. The β-barrel porins are an excellent example of this phenomenon with many sequence pairs sharing very low sequence identity (sometimes only ∼11%) based on structural alignment. These diverged sequences all share strongly conserved structural features that can be used to inform computational models. These features include: (1) even number of anti-parallel trans-membrane strands; (2) a relatively high abundance of small hydrophobic residues at the center of the trans-membrane strands facing the membrane; and (3) girdles of aromatic residues at the membrane interfaces [12], [19], [20].

Algorithms have been developed over the past decade to predict β-barrel secondary structure features using “rules” based on these conserved features with a varying degree of detail [21]–[24]. Some algorithms predict whether the query sequence more likely belongs to a membrane β-barrel versus a globular protein [25]–[27], while others predict the number (i.e. 8, 10 12, 16, 22-stranded) and position of membrane spanning β-strands [20], [28], [29].

To date, several groups have assigned secondary structure topology for MOMP of *Chlamydia muridarum* (previously called *Chlamydia trachomatis* mouse pneumonitis, MoPn) and *C. trachomatis* serovars D and F based on these algorithms, biochemical and immunological data [30]–[32]. These studies provide fairly similar assignments of the transmembrane strands; all suggest an overall 16-stranded topology, the models vary primarily in the length and placement of periplasmic turns and external loops with a few variations in positions of β-strands. However, these topologies cannot be directly translated into a 3D molecular model based on published 16-stranded trimeric β-barrels coordinates because the topology maps do not incorporate one or more of the structural elements of these 3D coordinates. For example, all known 16-stranded β-barrel porin crystal structures have an eyelet loop that folds into the pore center and is formed by the third loop [33]; the number of residues demarked as loop 3 in two of the three published topology maps are too short to form this conserved structural feature.

In this work, a recently developed suite of β-barrel topology prediction algorithms, TMBpro [29], combined with comparative homology building methods, and model refinement were used to develop a three-dimensional 16-stranded β-barrel homo-trimer model for the *C. trachomatis* MOMP protein. These methods and the resulting structures illustrate regions where the MOMP protein sequence is consistent with structural and functional features of 16-stranded templates, as well as regions that are structurally inconsistent with known templates. This enigma motivates further experimental structural studies of this important vaccine target.

## Results

### Prediction of β-barrel topology

**Figure 1:**
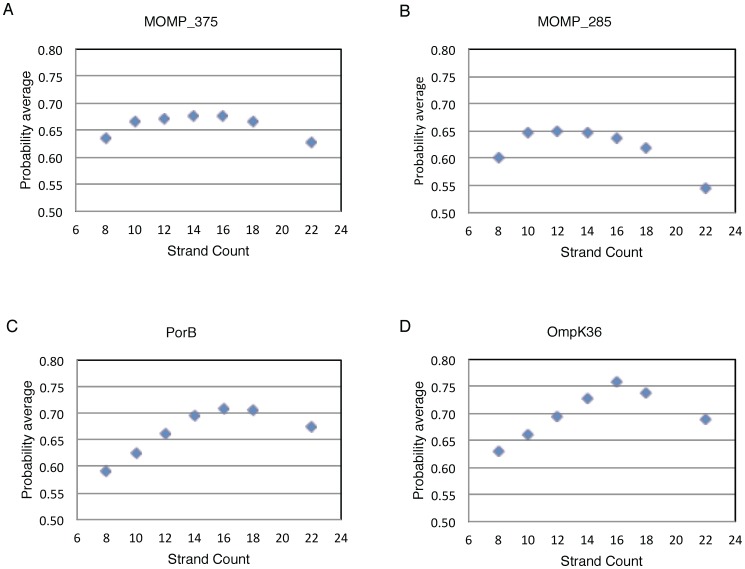
TMBpro β-barrel topology predictions. Normalized predicted probabilities for various strand models of (A) full length *C. trachomatis* serovar C sequence; (B) example output for *C. trachomatis* serovar C excluding a subset of residues that contains the leader sequence and all or parts of the four VDs: M1 – A23, A64 – P85, K152 – A158, T216 – I247, T282 – V310; (C) *Neissera meningitidis* PorB (3A2T.pdb protein sequence) [39]; and (D) *Klebsiella pneumonia* OmpK36 (1OSM.pdb protein sequence) [35].

**Figure 2:**
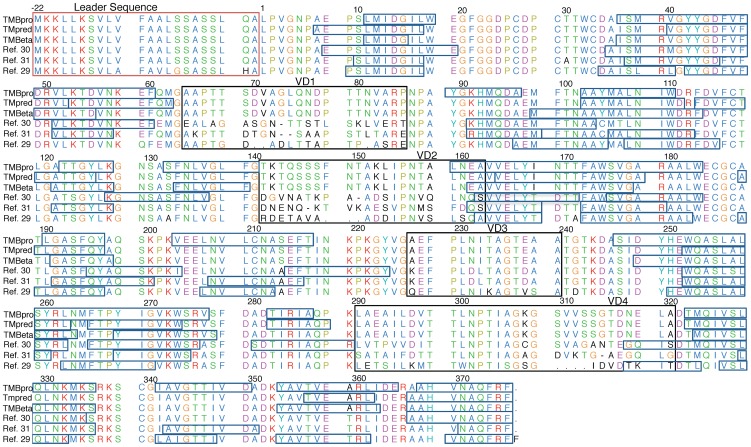
β-strand predictions mapped onto MOMP sequences. The output from β-strand prediction algorithms TMBpro, TMPRED and TMBBETA-NET (labeled pred1, pred2, and pred3, respectively) are delineated as blue boxes on the MOMP sequences from *C. trachomatis* serovar C. Previously reported β-strand predictions from 2D topology mapping for MOMP are shown for comparison on the sequences used in each publication, namely MOMP *C. trachomatis* serovar F [31] and serovar D [32] and *C. muridarum* [30]. Red and black boxes delineate the N-terminal leader sequence and 4 variable domains, respectively. Sequence alignments and illustration made with UCSF-Chimera visualization tools (http://www.cgl.ucsf.edu/chimera) [75].

A consensus sequence for human *C. trachomatis* serovar C MOMP, excluding the N-terminal leader sequence, was submitted to the TMBpro server [29] for initial predictions of overall β-barrel topology (Figure 1). The results assign fairly equal probability to the MOMP monomer subunit being comprised of 10-, 12-, 14-, 16- or 18- β–strands. Additionally, the TMBpro server predicts the probabilities of individual residues in transmembrane β-strands (*SI Table 1*). Figure 2 compares the predicted β-strand limits to those predicted using Pred-TMBB [22] and TMBeta-NET [26], [34] webservers and the previously published 2D topology maps for the MOMP porin. Several trends can be observed from this comparison: (1) the variable domain regions are predicted to be outside the trans-membrane β-strands; (2) all the algorithms predict similar residues to be in the first 2 and final β-strands; and (3) there is not a consensus for the limits of the remaining β-strands or, in some regions, whether a β-strand is present or not. Specifically, it is clear that all the algorithms suggest the presence of a long cysteine-containing loop spanning residues G20 to D34, but the Y89 to T100, F113– F139, V203 - I217, A280 – P288, and I341 – V355 sequences have little prediction consensus.

The TMBpro algorithm and other β-strand assignment algorithms predict several long strand sequences of 18 residues or more that are consistent with the long β-strands protruding from the extracellular membrane, as observed in 12- and 14-stranded barrels. However, comparisons of results across multiple algorithms illustrate that some of these long strands can also be predicted as two distinct strands with an intervening turn. Such a result is more consistent with shorter strand – turn – strand assignments observed in 16 -, 18- or 22-stranded barrels. Thus, depending upon where a given algorithm predicts the strand termini, there can be a significant difference in the overall topology predicted.

The topology predictions for two sequences from known crystal structures not included in the original TMBpro training sets are shown to compare the relative prediction accuracy, especially for β-barrels with twilight zone sequence identity. The *Neisseria meningitidis* porin, PorB [35], has less than 11% sequence identity to any of the crystal structures used in the original TMBpro training set. Similar to the topology probabilities observed for MOMP, several β-strand topologies had approximately equal probabilities (Figure 1C), however, the predictions are more distinguishable than those for MOMP. Another sequence, *Klebsiella pneumoniae* Omp36K [35], a porin that has 60% identity to one of the 16-stranded barrels used in the TMBpro training set has a much more distinct prediction for a 16-stranded topology (Figure 1D).

As transmembrane β-barrel prediction methods are also known to be sensitive to insertions/deletions of long extracellular sequences [23], MOMP sequences were submitted to these servers in several different forms, each varying by the exclusion of one or more VD. This procedure and the comparison of results from three algorithms, each generated using different prediction protocols, allowed us to evaluate the robustness of the predictions. Omission of various subsets of residues containing all or parts of the four VD shift the predicted probability for a particular β-barrel topology, but all such experiments produced nearly identical prediction of the residues involved in transmembrane β-strands (see Figure 1B for one such example).

### MOMP monomer models

**Figure 3:**
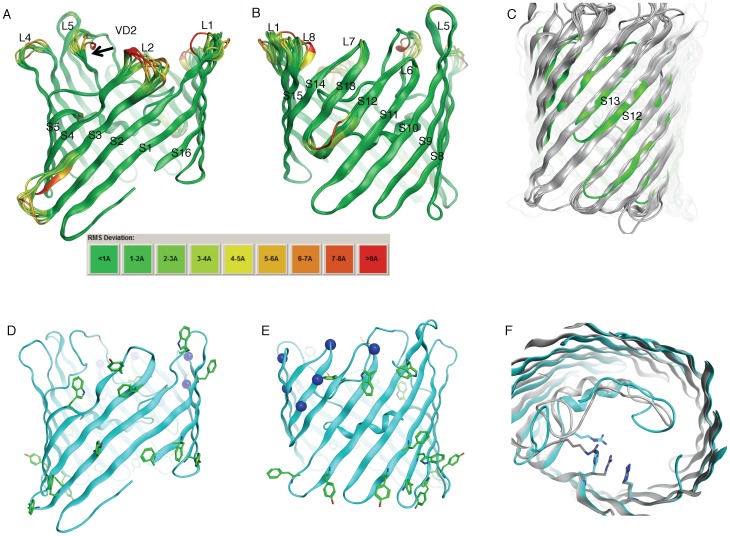
MOMP monomer model. (A, B) Backbone superpositions of the 10 lowest energy monomer structures. The ribbons illustrate the relative RMS deviations for the backbone heavy atoms to the average structure by color from low [75] to high (red). (C) The average backbone representation for the MOMP monomer [75] superposed onto backbone coordinates for crystal structures OmpF, OmpC [64], Omp36K [35], PhoE [76], PorB [39] and OprP [59] (gray ribbon). Superpositions were made using MOE, optimizing for structural and sequence alignments. (D, E) The MOMP monomer 16-stranded β-barrel with backbone in ribbon representation, aromatic girdle residues [75] and position of basic residue girdle (blue spheres), trimer interface and membrane interfaces, respectively. (F) The MOMP model, looking down the pore channel from the membrane exterior side (cyan ribbon) with putative anion pathway residues shown (cyan stick representation for R39, H92, R112) superposed on *E. coli* OmpF structure illustrating anion pathway residues (gray, 1HXX [73]).

Monomer modeling proceeded by assuming a 16-stranded β-barrel topology. Figure 3 panels A and B illustrate the 10 lowest energy 16-stranded model for MOMP. These models were generated through the iterative superposition of MOMP residues predicted as β-strand residues to the composite porin crystal structure template and refinement as described in Methods. These models represent ∼70% of the MOMP amino acid sequence.

**Figure 4:**
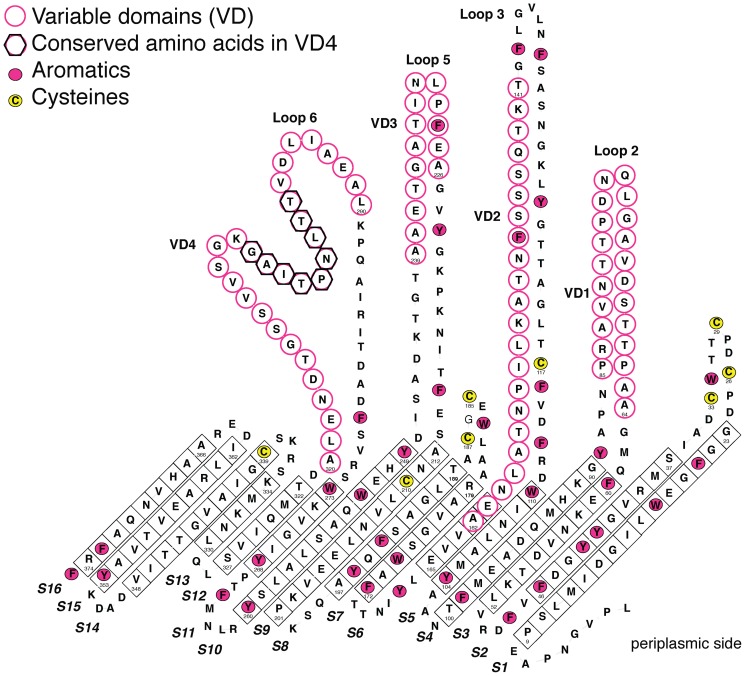
Predicted topology of the *C.* *trachomatis* MOMP serovar C monomer.

The final monomer models have >95% of the residues in the allowed Ramachandran values and where most of the outliers are found in loop junctions with the β-strands (outliers in 5 or more of the 10 lowest energy structures are Y104, I109, R112, S145, L182, A320, D321 and S335; see predicted topology map Figure 4). The limits of the 16 β-strands are P9 - G23, S37 - F46, L52 -F60, G90 - T100, Y104 - W110, E161 - E165, F172 - R179, T189 - A197, P201- A212, Y249 - Y260, Y268 - W273, T322 - S327, L330 - K334, C339 - V348, Y353 - I362, and A366 - R374 for the average structure. Given the uncertainty in the β-strand termini based on TMBpro predictions and the variation in template termini coordinates, these termini may differ up to +/− 3 residues; in fact, Kabsch & Sanders secondary structure calculations [36] assign alternate strand termini across the 10 lowest energy monomer structures for all β-strands except those of β -strands 6 and 7 (data not shown). Overall, the strand limits assigned superpose well with strand lengths defined by the composite template, as judged by the low RMSD in these regions (green, Figure 3A, 3B). Regions of higher RMSD (yellow – red) are limited to outer loops, the periplasmic turn between β- strands 2 and 3 and β-strands 12 and 13. Surprisingly external loops 5–7 converge to a single conformation for these structures.

Residues T322 to R336 have a high probability to be in the TM region of the pore by TMBpro (*SI Table 1*), with a clear decrease in probability for residues S338 – G340 and a strong probability for another TM β-strand starting at I341. According to the topology requirements of a 16-stranded β-barrel and the experimentally derived requirement to have VD4 (residues L290 – A320) externally exposed, residues T322 to R336 must span the membrane twice. Further limiting the assignment of this region to TM strands are the highly polar nature of residues K332, K334, S335, R336, K337 and S338, expected to be in a loop or turn outside of the membrane. Figure 3C shows the superposition of the average MOMP monomer model structure to six other 16-stranded β-barrel crystal structures. It illustrates that substituting another template structure for the hybrid template used will not solve this dilemma as the number of residues required to span β-strands 12 and 13 would be too short for any of these templates.

Aromatic girdles, a hallmark of membrane β-barrels, are most prominent in the MOMP β-strands comprising the trimer interface (Figure 3D) and around the periplasmic layer interface (Figure 3E). A clustering of charged lysine and arginine residues at the β-strand termini of β-strands 11, 13–16 may compensate for the lack of aromatic residues at the external membrane interface. The MOMP girdles are similarly located to polar girdles observed in other porins [12], [33] where these charged residues can provide anchors at the membrane interface because of their highly unfavorable partition energies into the membrane [37]. Yet, unique to MOMP is a sequence region assigned to β-strands 12–14 that is devoid of aromatic residues.

When the MOMP monomer model is aligned to a number of other 16-stranded porin structures, the MOMP sequences corresponding to β-strands 1–2 and 16 demonstrate the highest sequence identity (*SI Figure 1*). Specifically residues G15, G41 and G44 are on strands 1 and 2, and aromatic residues F373 and F375 are on strand 16. F375 participates in “aromatic rescue”, a structural feature in which the aromatic ring covers and stabilizes a glycine residue [20] on β-strand 1. This alignment also suggests that the MOMP pore contains a cluster of basic residues in the channel wall at positions R39, H92 and R112 (Figure 3F) that typically comprise the porin anion transport pathway [14], [38], [39]. The H92 residue in MOMP is typically an arginine in other porins. H92 may be doubly protonated and therefore, also carry a formal positive charge, alternatively, this position may be occupied by MOMP's K91 via a slightly different alignment or a β-bulge. Porin structures typically also have a conserved glutamate in the anion transport pathway in β-strand 3, and the MOMP model has N57 at this position (*SI Figure 1*). Although several attempts were made to align E59 to this position by readjustment of the alignment by two residues, these models placed a K58 (which is typically a hydrophobic residue in other porins) at the trimer interface.

### MOMP Trimer Model

The MOMP model trimer interface is comprised of β-strands 1–4 and 16. The average monomer-monomer surface area is 1316 Å^2^ and has exclusively hydrophobic inter-strand interactions. Typical barrel interfaces are slightly larger, ∼1400 Å^2^ and contain hydrogen bonding and/or salt bridge interactions in non-strand regions of the interface, in addition to the exclusively hydrophobic interactions between β-strands (data not shown). These differences between the MOMP model and other trimeric porins and the PISA algorithm low probability for protein-protein interface formation may be due to the omission of the loop region sequences from MOMP at β-strand termini where these types of interactions are typically found.

Recently, an algorithm has been developed by Naveed and colleagues that identifies weakly stable transmembrane β-strand regions in β-barrel porin structures [40]. These porin regions are correlated with protein-protein oligomerization interfaces and the per β-strand empirical energy values have been experimentally validated [41], [42]. Submission of our β-strand residue regions and the *C. trachomatis* serovar C amino acid sequence to the webserver for this algorithm identifies β-strands 1–5, 12–13 and 16 as relatively unstable and therefore candidate regions for oligomerization [43] (data not shown). Overall, the empirical energy profile calculated from our β-strands 1–5 and 16 closely match those of oligomeric β-barrel membrane protein profiles (H. Naveed, personal communication) and are consistent with oligomeric trimer formation at this interface.

**Figure 5:**
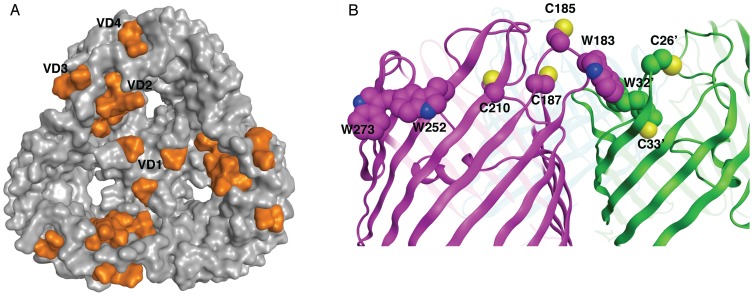
MOMP trimer structural features. (A) MOMP model surface, mapping of VD. Positions attaching the VDs to the barrel mapped (orange) onto the molecular surface of the MOMP trimer model. (B) MOMP loops 1 and 4 potential inter-monomer stabilizing contacts. Two of the three monomer β-barrels (green and magenta ribbon representation) illustrate the proximity of Loops 1 and 4. Their tryptophan and cysteine residues (space-filling atom representation) on neighboring trimer subunits are shown (C29 not modeled). Residues W252 and W273 are at the exterior membrane interface in the putative aromatic girdle.

Figure 5A illustrates a view of the trimer model from the perspective of the external membrane surface. Although the VDs are not modeled, the sequence termini leading to VDs 2, 3, and 4 appear in close proximity to one another, and the loops containing these residues may form a surface within each monomer. VD1 is located on Loop 2, a loop that is observed to form interactions between monomers in other trimeric porins. Figure 5A,B also shows that the VD1 sequences (Loop 2) within each monomer are in close proximity to each other at the trimer interface. Although *E. coli's* OmpF has a salt bridge in this “latching Loop 2” [44], there is no evidence that this is conserved in other trimeric porins or MOMP. Given the length of the omitted residues from Loop 2 of this model, the mapping of B-cell epitopes to this loop and the inter-monomer proximity of the three Loop 2 regions at the trimer interface, it is likely the Loop 2 region of each monomer combine to form a structural epitope that protrudes from the membrane surface. Conserved features also observed in the MOMP trimer are the Y89 residues from each monomer forming a ring at the top of the central core. In 16- and 18-stranded β-barrels these tyrosine hydroxyl groups coordinate bound water.

## Discussion

The development of a 3D structural model with no detectable sequence similarity to a protein with experimentally determined structure is non-trivial and can only be accomplished with certainty in protein families with highly conserved structural features (e.g. porins). Highly conserved structural features observed among porin β-barrels have led to the development of algorithms to detect and predict the presence of β-strands and the overall topology of membrane porins. However, these algorithms are not without pitfalls. As shown with the MOMP example, there can be ambiguity in delimiting the number of β-strands. In addition, as more membrane bound β-barrel crystal structures are determined, exceptions to the list of 10 explicit rules governing porin structure, as outlined by Schulz [12], are found. For example, in recent structures for mitochondrial protein VDAC, the β-barrel is comprised of 19 β-strands (2JK4) [45], [46], where it was previously considered that β-barrels were comprised solely of an even number of strands. In another recent structure, the barrel is made up of 3 subunits, each making up 4 strands of the 12-stranded barrel [47], illustrating another unique barrel fold.

Despite these challenges and the ambiguity in the TMBpro overall topology prediction for the MOMP sequence in the C-terminal region, we proceeded to construct and assess a 16-stranded β-barrel model based on previously published experimental evidence. The most convincing experimental evidence is the detection of oligomeric structures for MOMP [15], which would exclude all currently solved 14-stranded barrels as templates and be more consistent with a 16- or 18-stranded barrel. Furthermore, the size dependence for measured transport rates of various sugars in MOMP containing micelles [15] suggest a MOMP pore size similar to that of 16-stranded OprF β-barrel. Electron microscopy for *C. trachomatis* elementary bodies results in a measured outer membrane width of 20 Å [48], consistent with other known porin widths.

The 3D MOMP model that was constructed contains several features common to other 16-stranded trimer structures. These features include: (1) basic residues at key transport positions in the pore channel (Figure 3F); (2) aromatic rings at the membrane boundaries (Figure 3D, 3E); and (3) highly conserved residues in β-strands comprising the trimer interface. Furthermore, relative secondary structure distribution for our final MOMP model is similar to that measured for native MOMP by circular dichroism: ∼38% β-strand and 3% αlpha-helix versus 38–44% β-strand and ∼5% alpha-helix, respectively [15].

The *Chlamydia* MOMP sequence has 8–10 cysteine residues, compared to other porin proteins, which typically have none. Eight of these cysteines, C26, C29, C33, C117, C185, C187, C210 and C339, are essentially completely conserved across serovars [49] and six are conserved across various MOMP orthologs; thus there appears to be evolutionary pressure to maintain cysteines at these particular positions. Additionally, MOMP requires some disulfide bonds to function as a pore [16]. The function of these cysteine residues has been postulated to provide stability to the outer membrane [50]–[53] by forming either intra-monomer and/or inter-monomer disulfide bonds to compensate for the loss of a peptidoglycan stabilized membrane [53]–[55]. Others suggest that at least some cysteine residues face the periplasm where they can form disulfide bridges with other proteins as part of a larger, stabilizing complex [48]. More recently, for other porins, such exposed cysteines have been postulated to be involved in the folding process with accessory proteins in the cytoplasm [56]. The MOMP model presented here supports the possibility of the first hypothesis, as the cysteine residues are found at the external membrane surface and primarily in the external loops, Figure 4. Three of the eight cysteine residues, C26, C29 and C33, are found in Loop 1. These may form an intra-monomer disulfide bond, similar to that seen in Loop 1 of 18-stranded barrels for *E. coli* and *S. enterica* maltoporin (1AF6 and 2MPR, respectively) [57], [58]; or, alternately, the proximity of MOMP's Loop 1 to Loop 4 of the neighboring monomer, containing C185 and C187, may allow inter-monomer disulfide bond formation. Cysteine residues that protrude into the lipid bilayer have been exhibited by other β-barrel proteins (e.g. VDAC1 [46]), and this cannot yet be ruled out as a possibility for MOMP. Mapping of disulfide bonds in MOMP has yielded conflicting results [32], [53]

The tryptophan residues found in the aromatic girdle and at the trimer interface may provide additional stability to the MOMP trimer. While 16-stranded porins typically have 3–4 tryptophan residues, *P. aeruginosa* OprP has 10 (2O4V) [59] and *C. trachomatis* MOMP has 7. Tryptophan has the highest stabilization energy contribution of any residue for membrane proteins and is known to play a key role in the proper folding of membrane proteins [60]–[62]. Most of the MOMP tryptophan residues are observed in the aromatic girdle regions of the protein and thus may provide critical stabilizing interactions at the interface of membrane surface. An additional tryptophan residue on Loop 1 (W32) in our model provides key packing interactions with putative cysteine disulfide bonds and a tryptophan in Loop 4 (W183) (Figure 5B). A notable outcome of the three-dimensional model is the finding that Loop 1 and Loop 4 may be involved in stabilizing the trimer interface, as these loops have not been previously identified as being involved in immunogenic epitopes [49]. Loops 1 and 4 from neighboring monomers are often proximal in experimentally derived crystal structures for porin trimers [39], [59], [63], [64]. The residues forming inter-monomer contacts in this region are predominately hydrophilic, providing hydrogen bonds or electrostatic interactions. Disulfide bond and aromatic pi stacking interactions between these loops present novel structural features for trimer porin stabilization.

The MOMP model presented here exhibits several key structural features consistent with experimental evidence for this porin. However, the inability to resolve the structure features of residues 320–334 with what is known about other porins and the necessity of this region to span the trans-membrane suggest MOMP has some as of yet unknown unique structural characteristics for a bacterial porin. This unique nature of MOMP is not likely to be merely a shortcoming of trying to model a twilight zone protein, but rather reflects constraints that cannot be resolved based on the necessity for VD4 to be external to the pore and the unlikelihood that a sequence of charged residues K334 – S339 form a trans-membrane strand. Baehr et al. [65] proposed that the TTLNPTIAGK region in VD4, that is conserved among all the human *C. trachomatis* serovars, might be folded to form a practically inaccessible loop that can served as a site for attachment to the eukaryotic cell. This constraint may explain some of the difficulties we have encountered modeling this region of the protein. The possibility that MOMP exists in multiple conformations, similar to the major porin OprF of *P. aeruginosa*, cannot be ruled out [56]. Unlike *P. aeruginosa*, *Chlamydia* do not have a peptidoglycan layer, although other proteins (in particular the cysteine-rich proteins 15 and 60-kDa) may play a compensatory biological role and MOMP may interact with them. It seems likely that MOMP adopts multiple conformations during the developmental cycle of *Chlamydia* and, in addition, that MOMP cysteine residues interact with other periplasmic proteins (again the 15 and 60 kDa cysteine-rich proteins) as indicated by Sugawara et al [56]. Indeed, the inability of current algorithms to assign significant probabilities to the C-terminus strand assignments for MOMP (Figures 1 and 2) could arise from the multiple conformations, on which the prediction algorithms have not been trained.

While methods other than template based homology modeling exist for developing structural models, namely *ab initio* folding through constrained simulated annealing [66], perhaps including constraints based on residue covariance across orthologs [67], [68], MOMP is at the upper limit of protein sequence length tried with covariance methods. Additionally, the number of MOMP orthologs sequenced is comparatively low for developing these constraints and recent attempts for covariance constraints on β-barrel proteins have given results with mixed success [68]. Overall, this 3D MOMP model represents the best case for incorporation of current experimentally derived knowledge for MOMP and structural knowledge for bacterial β-barrels. Although the model is speculative in relation to a comparative model built with higher sequence identity to 16-stranded β-barrels, it does illustrate intriguing unique features that motivate further structural and functional investigations for this important vaccine target.

## Materials and Methods

**Figure 6:**
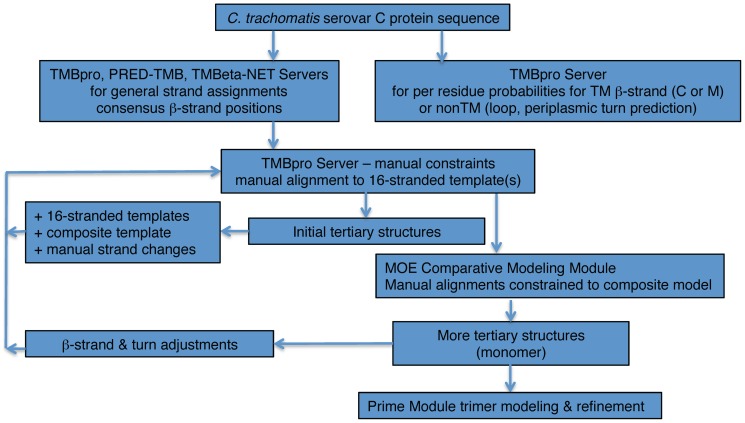
MOMP model construction flowchart.

Figure 6 provides a Flowchart for the data input and the iterative model building procedure described below.

### TMBpro Prediction for Barrel Topology and β-strand probabilities

A *C. trachomatis* serovar C majority consensus sequence was constructed from all serovar C *omp1* gene sequences available in GenBank on December 2, 2011. This consensus sequence is identical at the amino acid level to that of isolate CS-362-07 (GenBank accession DQ116399). This sequence, excluding the first 22 residues, was submitted to TMBpro server (http://www.igb.uci.edu/~baldig/tmb.html) [29]. Additional submissions were made excluding residues (i) A226–A238 and L290 - D316, (ii) A64–A 83 and L290 – D316 or (iii) A64 – P85, K152 – A158, T216 – I247, T282 – V310. Additional 16-stranded β-barrel crystal structure coordinates were added to the TMBpro template database manually for comparison of automated 3D MOMP output by the server; pdb codes 1OSM [35], 1PHO [69] and 2J1N [64] and a composite template based on *E. coli* OmpF and *C. acidovorans* Omp32; residues A1 – R100 and F267 – F340 from 2OMF [69] and residues L94 – G250 from 1E54 [38]. The algorithm's refinement steps toward reducing β-carbon clashes between neighboring residues on the barrel strands [29] were considered and these β-strand to template sequence alignments were used as starting points for further refinement using MOE (see below).

The *C. trachomatis* serovar C majority consensus sequence described above was also submitted to servers for PRED-TMBB (http://biophysics.biol.uoa.gr/PRED-TMBB/) [22] and TMBbeta-NET (http://psfs.crc.jp/tmbeta-net/) [26]. A sequence omitting the majority of variable domain residues (Q61 – P85, T141 – A162, A226 – A238, L290 – A320) [70] was also submitted to each server.

### Porin template selection

Structural template selection was based on the (i) the lowest overall 3D model search score for the four models built on 16-stranded templates used by TMBpro and (ii) accommodation of the putative MOMP Loop 3 and VD2. Table 1 lists initial search scores for 3D MOMP models built by TMBpro protocols on four different 16-stranded β-barrel templates prior to any manual modifications in β-strand assignment. MOMP had the lowest search scores with *E. coli* OmpF and *Comamonas acidovorans* Omp32 porin [38] template structures.

**Table 1:** TMBpro Model Search Energies by Structural Template.

Template Porin	pdb code	TMBPro Model Score^1^
*E. coli* OmpF	2OMF	25,743
*C. acidovarans* Omp32	1E54	26,845
*R. capsulatus* Porin	POR	30,812
*R. pseudomonas* Porin	1PRN	33,558
OmpF-Omp32 Hybrid	2OMF & 1E54	15,129

1 TMBpro search scores calculated as described in Randall et al. [29]. The structure with the lowest score (the best structure) of the 50 structures calculated for each template is reported.

Special consideration was needed for modeling the putative Loop 3 region, which forms a pore filling eyelet in all solved 16-stranded porin structures [12], [71]. MOMP residues from ∼W110 to F139 were predicted to fold into the pore based on their C-terminal proximity to β-strand 5 and the variable and weak probabilities to form β-strands (*SI Table 1*, *Figure 2*). VD2 is immediately C-terminal to F139 and known to contain epitopes, thus is unlikely that this domain would also be buried within the channel. Evaluation of structures for nine known unique 16-stranded β-barrel porins revealed two examples where the C-terminal region of the eyelet loop formed an additional loop protruding toward the external membrane surface, e.g., *C. acidovorans* Omp32 (1E54) [38] and *Rhodopseudomonas blastica* porin (3PRN) [72], and thus this may also be the case for MOMP. Based on the relatively low TMBpro search scores for MOMP built on the Omp porin templates, where lower scores are considered to be favorable, a composite MOMP template was built based on OmpF that incorporated the eyelet loop in the Omp32 structure and its C-terminal protrusion. In addition to Loop 3, β-strands 6 through 12 of Omp32 were included in the hybrid template because the Loop 3 protrusion in Omp32 would overlap with OmpF loop coordinates protruding from its strands 6–12. This template reduced potential packing clashes between the channel wall and internal Loop 3. Basing C-terminal strands 13–16 on the OmpF template resulted in a consistent trimer interface, packing interactions and closure of the barrel with the N-terminal strands. Ultimately, building the MOMP sequence on this composite template and guiding manual strand position constraints for β-strands 1–6, 15 and 16 resulted in a significantly lower search score compared to prior templates (Table 1).

### Monomer Model Construction

Monomer models were built with the homology module in MOE (Molecular Operating Environment, 2011.10, Chemical Computing Group Inc., 1010 Sherbooke St. West, Suite #910, Montreal, QC, Canada H3A 2R7, 2011). Default settings, including automated disulfide bond detection were set to give 25 “medium” refined structures per alignment and scored based on contact energy, packing score and MOE's GB/VI energy. Manually derived sequence alignments were maintained between each template and MOMP sequence during model building by constraining the putative β-strand regions (17–20 constraints per run). This limited number of constraints allows some modifications in β-strand alignment thus different model outcomes. The models were analyzed for relative energies and protein geometry to minimize the number of outlier residues for Ramachandran values, bond lengths, angles, and rotameric states.

The TMBpro server output includes per residue predictions for the query sequence into three bins; membrane-facing, channel-facing or non-transmembrane. Adjustment to the automated TMBpro β-strand to template alignments were made after considering the following criteria: (i) consensus of β-strand residues by various algorithms, Figure 2, (ii) comparison with β-strand positions in published 2D topology maps, Figure 2, (iii) the probabilities for channel facing (C) and membrane-facing (M) (*SI Table 1*), and (iv) aromatic residue positions consistent with the putative membrane interface.

The probabilities for channel facing vs. membrane-facing residues provided a crucial criterion for assessing specific residue alignments to the templates, especially where residues would not be considered entirely hydrophobic. For example, lysine or arginine residues are generally considered unfavorable as membrane-facing residues due to their charged functional groups, yet there is precedence for these residues to “snorkel” [20] at β-strand termini. Snorkeling involves the positioning of long hydrophobic side chains inside the membrane and charged groups at the polar membrane interface. Additionally, although threonine residues are not hydrophobic, they are found to be membrane facing more often than expected. Deviations from the concept of merely alternating hydrophobic and hydrophilic residue patterns in trans-membrane β-strands has been noted by Wimley, et al. [19], who found that analyzing abundance patterns for residues enriched at β-strand centers or membrane limits may provide a more reliable prediction scheme for residue orientation.

### Homo-Trimer Construction and Analysis

β-strand limits for the MOMP monomer were used for align input files to the Prime homology module (Prime, version 3.0, Schrödinger, Inc., New York, NY, 2011) the module used to build the MOMP homo-trimer model. A remodeled OmpF-Omp32 hybrid template based on the high resolution *E. coli* OmpF coordinates 1HXX [73], and superimposed three copies for the monomer template to each subunit of the *E. coli* OmpF trimer to served as the input template for trimeric MOMP model. The higher resolution OmpF coordinates have fewer contact errors at the trimer interface and hence propagates fewer errors to the MOMP model. Each MOMP model monomer has residues L1 – C26, T31 – G63, P87 – S147, P155 – F215, D248 – R275, A320 – F375.

### Structural Analyses

Final models were submitted to the PISA server for trimer interface surface area calculation and analysis (http://www.ebi.ac.uk/msd-srv/prot_int/pistart.html) [74]. Note that the surface area is only counted once for each interface.

## Supporting Information

Figure S1 **MOMP sequence alignment based on structural alignment to other 16-stranded porin structures.** β-strand positions (blue arrows), porin residues described as conserved by Tanabe et al. [39] (yellow) and the hybrid template splicing positions (orange line) are shown. Variable domain positions (truncated in some cases) are indicated in red, position of eyelet loop shown in green.
(TIFF)

Model S1 C_trachomatis_serovarC_MOMPmodel_final_May2013
(PDB)

Table S1 **Per residue probabilities for transmembrane strand placement, TMBpro (and 2D Topology Assignment).** Variable domains indicated in orange boxes.
(PDF)
