# Supplementary figures and images for: A 3-Dimensional Trimeric β-Barrel Model for Chlamydia MOMP Contains Conserved and Novel Elements of Gram-Negative Bacterial Porins
Source: PLoS One. 2013 Jul 25;8(7):e68934. doi: 10.1371/journal.pone.0068934 (PMC3723809; doi:10.1371/journal.pone.0068934)

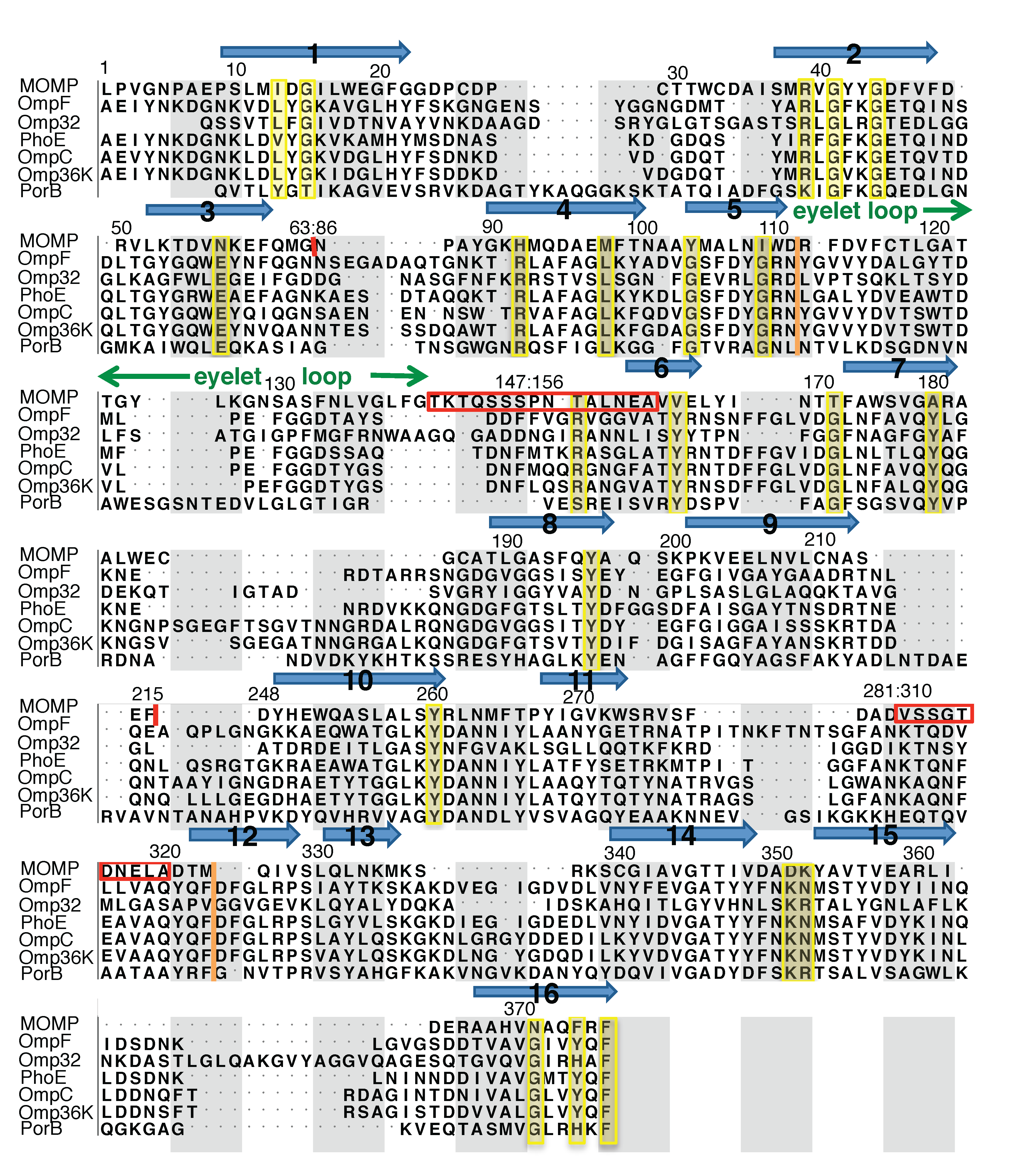

Supplement: Figure S1 — MOMP sequence alignment based on structural alignment to other 16-stranded porin structures. β-strand positions (blue arrows), porin residues described as conserved by Tanabe et al. [39] (yellow) and the hybrid template splicing positions (orange line) are shown. Variable domain positions (truncated in some cases) are indicated in red, position of eyelet loop shown in green. (TIFF) [file pone.0068934.s001.tiff]
