# Supplementary material for: A 3-Dimensional Trimeric β-Barrel Model for Chlamydia MOMP Contains Conserved and Novel Elements of Gram-Negative Bacterial Porins
Source: PLoS One. 2013 Jul 25;8(7):e68934. doi: 10.1371/journal.pone.0068934 (PMC3723809; doi:10.1371/journal.pone.0068934)
Supplement: Table S1 — Per residue probabilities for transmembrane strand placement, TMBpro (and 2D Topology Assignment). Variable domains indicated in orange boxes. (PDF) [file pone.0068934.s003.pdf]

Idx	MOMP Serovar C	2-class	non-TM	TM	3-class	non-TM	Channel	Membrane
1	L		0.877	0.123		0.881	0.033	0.086
2	P		0.936	0.064		0.954	0.031	0.015
3	V		0.945	0.055		0.957	0.016	0.027
4	G		0.972	0.028		0.976	0.015	0.009
5	N		0.981	0.019		0.975	0.01	0.016
6	P		0.951	0.049		0.968	0.023	0.009
7	A		0.958	0.042		0.911	0.021	0.068
8	E		0.942	0.058		0.828	0.159	0.013
9	P		0.891	0.109		0.778	0.02	0.202
10	S		0.617	0.383		0.454	0.518	0.028
11	L		0.238	0.762		0.372	0.033	0.595
12	M		0.179	0.821		0.352	0.609	0.039
13	I		0.21	0.79		0.428	0.043	0.529
14	D		0.273	0.727		0.527	0.436	0.036
15	G		0.505	0.495		0.704	0.023	0.273
16	I		0.256	0.744		0.571	0.332	0.098
17	L		0.277	0.723		0.691	0.08	0.229
18	W		0.222	0.778		0.603	0.231	0.166
19	E		0.537	0.463		0.831	0.095	0.074
20	G		0.836	0.164		0.94	0.027	0.033
21	F		0.853	0.147		0.931	0.039	0.03
22	G		0.922	0.078		0.963	0.02	0.017
23	G		0.966	0.034		0.975	0.012	0.013
24	D		0.963	0.037		0.973	0.013	0.013
25	P		0.973	0.027		0.983	0.01	0.007
26	C		0.976	0.024		0.976	0.01	0.013
27	D		0.983	0.017		0.983	0.011	0.006
28	P		0.981	0.019		0.982	0.008	0.01
29	C		0.95	0.05		0.951	0.036	0.012
30	T		0.904	0.096		0.872	0.028	0.101
31	T		0.819	0.181		0.784	0.185	0.031
32	W		0.726	0.274		0.684	0.051	0.264
33	C		0.661	0.339		0.725	0.229	0.046
34	D		0.555	0.445		0.611	0.04	0.349
35	A		0.286	0.714		0.407	0.541	0.052
36	I		0.081	0.919		0.074	0.029	0.897
37	S		0.04	0.96		0.046	0.937	0.017
38	M		0.02	0.98		0.016	0.012	0.972
39	R		0.016	0.984		0.015	0.972	0.013
40	V		0.018	0.982		0.01	0.012	0.978
41	G		0.029	0.971		0.019	0.967	0.013
42	Y		0.024	0.976		0.016	0.015	0.969

43	Y	0.03	0.97	0.017	0.958	0.025
44	G	0.201	0.799	0.068	0.022	0.909
45	D	0.166	0.834	0.078	0.893	0.029
46	F	0.165	0.835	0.054	0.022	0.924
47	V	0.098	0.902	0.063	0.912	0.026
48	F	0.273	0.727	0.155	0.039	0.806
49	D	0.232	0.768	0.239	0.726	0.035
50	R	0.205	0.795	0.165	0.045	0.79
51	V	0.151	0.849	0.168	0.794	0.038
52	L	0.203	0.797	0.174	0.025	0.801
53	K	0.203	0.797	0.131	0.834	0.036
54	T	0.272	0.728	0.166	0.027	0.807
55	D	0.503	0.497	0.364	0.613	0.023
56	V	0.627	0.373	0.476	0.024	0.5
57	N	0.689	0.311	0.601	0.376	0.023
58	K	0.529	0.471	0.463	0.025	0.512
59	E	0.264	0.736	0.281	0.673	0.046
60	F	0.286	0.714	0.321	0.045	0.635
61	Q	0.258	0.742	0.359	0.597	0.043
62	M	0.438	0.562	0.461	0.05	0.489
63	G	0.756	0.244	0.784	0.18	0.036
64	A	0.789	0.211	0.753	0.045	0.202
65	A	0.86	0.14	0.849	0.121	0.029
66	P	0.907	0.093	0.903	0.017	0.08
67	T	0.901	0.099	0.844	0.12	0.036
68	T	0.93	0.07	0.933	0.019	0.048
69	S	0.924	0.076	0.936	0.04	0.023
70	D	0.898	0.102	0.936	0.03	0.033
71	V	0.881	0.119	0.92	0.037	0.043
72	A	0.879	0.121	0.916	0.046	0.037
73	G	0.906	0.094	0.925	0.034	0.041
74	L	0.866	0.134	0.895	0.054	0.051
75	Q	0.854	0.146	0.892	0.057	0.052
76	N	0.884	0.116	0.899	0.055	0.045
77	D	0.912	0.088	0.911	0.037	0.052
78	P	0.915	0.085	0.913	0.057	0.03
79	T	0.909	0.091	0.828	0.04	0.132
80	T	0.896	0.104	0.838	0.131	0.032
81	N	0.877	0.123	0.772	0.035	0.193
82	V	0.865	0.135	0.813	0.158	0.029
83	A	0.903	0.097	0.868	0.026	0.107
84	R	0.918	0.082	0.903	0.078	0.019
85	P	0.894	0.106	0.867	0.028	0.105
86	N	0.925	0.075	0.895	0.077	0.028
87	P	0.761	0.239	0.763	0.056	0.181
88	A	0.555	0.445	0.512	0.303	0.185
89	Y	0.362	0.638	0.399	0.188	0.413
90	G	0.528	0.472	0.489	0.296	0.215
91	K	0.297	0.703	0.244	0.287	0.468

92	H	0.311	0.689	0.46	0.242	0.298
93	M	0.437	0.563	0.538	0.245	0.217
94	Q	0.589	0.411	0.677	0.107	0.216
95	D	0.694	0.306	0.832	0.101	0.067
96	A	0.79	0.21	0.865	0.055	0.08
97	E	0.825	0.175	0.868	0.081	0.051
98	M	0.692	0.308	0.811	0.096	0.093
99	F	0.528	0.472	0.735	0.088	0.178
100	T	0.372	0.628	0.639	0.237	0.124
101	N	0.674	0.326	0.768	0.052	0.179
102	A	0.346	0.654	0.546	0.346	0.108
103	A	0.161	0.839	0.277	0.122	0.601
104	Y	0.068	0.932	0.114	0.781	0.105
105	M	0.07	0.93	0.089	0.056	0.855
106	A	0.069	0.931	0.092	0.865	0.043
107	L	0.076	0.924	0.151	0.053	0.796
108	N	0.179	0.821	0.348	0.58	0.072
109	I	0.128	0.872	0.307	0.055	0.638
110	W	0.157	0.843	0.38	0.547	0.073
111	D	0.442	0.558	0.628	0.06	0.312
112	R	0.659	0.341	0.774	0.176	0.049
113	F	0.775	0.225	0.723	0.044	0.233
114	D	0.802	0.198	0.702	0.222	0.076
115	V	0.736	0.264	0.601	0.085	0.314
116	F	0.693	0.307	0.636	0.291	0.073
117	C	0.665	0.335	0.581	0.063	0.356
118	T	0.485	0.515	0.463	0.472	0.065
119	L	0.665	0.335	0.509	0.031	0.46
120	G	0.82	0.18	0.739	0.232	0.029
121	A	0.607	0.393	0.504	0.035	0.461
122	T	0.353	0.647	0.345	0.582	0.073
123	T	0.54	0.46	0.579	0.089	0.332
124	G	0.482	0.518	0.593	0.235	0.171
125	Y	0.164	0.836	0.322	0.255	0.423
126	L	0.157	0.843	0.345	0.315	0.34
127	K	0.28	0.72	0.438	0.245	0.317
128	G	0.646	0.354	0.811	0.086	0.103
129	N	0.69	0.31	0.786	0.097	0.116
130	S	0.574	0.426	0.688	0.211	0.101
131	A	0.504	0.496	0.602	0.122	0.277
132	S	0.39	0.61	0.496	0.405	0.1
133	F	0.307	0.693	0.453	0.09	0.457
134	N	0.31	0.69	0.502	0.411	0.087
135	L	0.084	0.916	0.245	0.116	0.639
136	V	0.055	0.945	0.189	0.646	0.166
137	G	0.088	0.912	0.329	0.185	0.486
138	L	0.047	0.953	0.199	0.52	0.281
139	F	0.094	0.906	0.312	0.246	0.443
140	G	0.405	0.595	0.74	0.148	0.112

141	T	0.428	0.572	0.733	0.083	0.184
142	K	0.569	0.431	0.798	0.124	0.078
143	T	0.655	0.345	0.772	0.076	0.152
144	Q	0.711	0.289	0.797	0.106	0.097
145	S	0.64	0.36	0.734	0.146	0.12
146	S	0.596	0.404	0.718	0.11	0.172
147	S	0.673	0.327	0.748	0.159	0.093
148	F	0.703	0.297	0.789	0.073	0.138
149	N	0.754	0.246	0.833	0.095	0.072
150	T	0.784	0.216	0.85	0.065	0.085
151	A	0.788	0.212	0.836	0.077	0.088
152	K	0.799	0.201	0.856	0.089	0.054
153	L	0.743	0.257	0.808	0.076	0.116
154	I	0.73	0.27	0.766	0.132	0.102
155	P	0.756	0.244	0.859	0.049	0.092
156	N	0.799	0.201	0.828	0.113	0.059
157	T	0.255	0.745	0.435	0.09	0.475
158	A	0.124	0.876	0.292	0.604	0.104
159	L	0.062	0.938	0.224	0.211	0.564
160	N	0.057	0.943	0.205	0.524	0.271
161	E	0.066	0.934	0.205	0.374	0.42
162	A	0.067	0.933	0.239	0.266	0.496
163	V	0.026	0.974	0.069	0.74	0.191
164	V	0.019	0.981	0.039	0.133	0.827
165	E	0.029	0.971	0.095	0.863	0.042
166	L	0.033	0.967	0.077	0.068	0.855
167	Y	0.06	0.94	0.157	0.753	0.09
168	I	0.137	0.863	0.273	0.049	0.678
169	N	0.432	0.568	0.553	0.416	0.031
170	T	0.347	0.653	0.23	0.037	0.733
171	T	0.224	0.776	0.197	0.771	0.033
172	F	0.259	0.741	0.116	0.015	0.869
173	A	0.098	0.902	0.055	0.93	0.015
174	W	0.044	0.956	0.019	0.012	0.97
175	S	0.053	0.947	0.023	0.967	0.01
176	V	0.064	0.936	0.019	0.01	0.971
177	G	0.216	0.784	0.055	0.933	0.012
178	A	0.159	0.841	0.041	0.011	0.948
179	R	0.151	0.849	0.043	0.944	0.014
180	A	0.277	0.723	0.071	0.014	0.915
181	A	0.314	0.686	0.12	0.862	0.019
182	L	0.299	0.701	0.186	0.026	0.787
183	W	0.295	0.705	0.227	0.706	0.067
184	E	0.667	0.333	0.607	0.044	0.348
185	C	0.782	0.218	0.722	0.245	0.034
186	G	0.922	0.078	0.785	0.015	0.2
187	C	0.742	0.258	0.488	0.475	0.037
188	A	0.638	0.362	0.248	0.023	0.729
189	T	0.286	0.714	0.064	0.916	0.019

190	L	0.392	0.608		0.089	0.013	0.898
191	G	0.459	0.541		0.128	0.859	0.014
192	A	0.127	0.873		0.038	0.009	0.953
193	S	0.047	0.953		0.015	0.976	0.009
194	F	0.035	0.965		0.013	0.012	0.975
195	Q	0.022	0.978		0.008	0.976	0.016
196	Y	0.036	0.964		0.02	0.024	0.956
197	A	0.124	0.876		0.047	0.914	0.038
198	Q	0.448	0.552		0.303	0.087	0.61
199	S	0.718	0.282		0.568	0.356	0.076
200	K	0.871	0.129		0.758	0.074	0.168
201	P	0.911	0.089		0.87	0.049	0.081
202	K	0.77	0.23		0.695	0.123	0.182
203	V	0.444	0.556		0.592	0.224	0.184
204	E	0.401	0.599		0.565	0.255	0.179
205	E	0.287	0.713		0.448	0.429	0.123
206	L	0.143	0.857		0.445	0.173	0.382
207	N	0.217	0.783		0.473	0.217	0.31
208	V	0.078	0.922		0.345	0.301	0.354
209	L	0.091	0.909		0.351	0.31	0.339
210	C	0.2	0.8		0.506	0.355	0.139
211	N	0.444	0.556		0.597	0.055	0.349
212	A	0.405	0.595		0.619	0.332	0.049
213	S	0.431	0.569		0.324	0.064	0.612
214	E	0.238	0.762		0.104	0.843	0.053
215	F	0.209	0.791		0.085	0.027	0.888
216	T	0.098	0.902		0.052	0.921	0.027
217	I	0.244	0.756		0.232	0.038	0.731
218	N	0.385	0.615		0.413	0.526	0.062
219	K	0.5	0.5		0.606	0.049	0.345
220	P	0.531	0.469		0.792	0.138	0.069
221	K	0.606	0.394		0.744	0.087	0.17
222	G	0.65	0.35		0.802	0.077	0.121
223	Y	0.332	0.668		0.574	0.268	0.157
224	V	0.361	0.639		0.559	0.183	0.258
225	G	0.707	0.293		0.769	0.158	0.072
226	A	0.739	0.261		0.793	0.063	0.144
227	E	0.812	0.188		0.82	0.152	0.027
228	F	0.905	0.095		0.899	0.022	0.079
229	P	0.927	0.073		0.936	0.046	0.018
230	L	0.925	0.075		0.916	0.019	0.065
231	N	0.911	0.089		0.928	0.053	0.019
232	I	0.883	0.117		0.867	0.033	0.099
233	T	0.829	0.171		0.831	0.134	0.035
234	A	0.884	0.116		0.869	0.037	0.094
235	G	0.898	0.102		0.913	0.054	0.033
236	T	0.792	0.208		0.786	0.082	0.132
237	E	0.84	0.16		0.827	0.099	0.074
238	A	0.84	0.16		0.813	0.084	0.103

239	A	0.83	0.17	0.831	0.067	0.102
240	T	0.779	0.221	0.784	0.131	0.085
241	G	0.827	0.173	0.836	0.048	0.117
242	T	0.733	0.267	0.772	0.147	0.08
243	K	0.71	0.29	0.745	0.064	0.191
244	D	0.619	0.381	0.629	0.296	0.075
245	A	0.412	0.588	0.487	0.088	0.425
246	S	0.137	0.863	0.193	0.655	0.152
247	I	0.166	0.834	0.274	0.206	0.52
248	D	0.125	0.875	0.246	0.396	0.358
249	Y	0.077	0.923	0.263	0.447	0.291
250	H	0.05	0.95	0.087	0.068	0.844
251	E	0.092	0.908	0.062	0.911	0.027
252	W	0.042	0.958	0.017	0.014	0.969
253	Q	0.035	0.965	0.016	0.973	0.011
254	A	0.05	0.95	0.013	0.01	0.977
255	S	0.114	0.886	0.027	0.963	0.01
256	L	0.083	0.917	0.019	0.008	0.972
257	A	0.041	0.959	0.014	0.975	0.011
258	L	0.023	0.977	0.01	0.009	0.981
259	S	0.02	0.98	0.014	0.977	0.009
260	Y	0.023	0.977	0.02	0.013	0.967
261	R	0.024	0.976	0.019	0.961	0.02
262	L	0.033	0.967	0.054	0.022	0.924
263	N	0.09	0.91	0.139	0.82	0.041
264	M	0.085	0.915	0.23	0.049	0.721
265	F	0.044	0.956	0.196	0.622	0.182
266	T	0.048	0.952	0.163	0.32	0.517
267	P	0.051	0.949	0.225	0.397	0.379
268	Y	0.029	0.971	0.09	0.341	0.569
269	I	0.021	0.979	0.068	0.477	0.455
270	G	0.039	0.961	0.111	0.475	0.414
271	V	0.016	0.984	0.069	0.37	0.561
272	K	0.019	0.981	0.051	0.589	0.359
273	W	0.025	0.975	0.065	0.392	0.543
274	S	0.081	0.919	0.248	0.356	0.396
275	R	0.287	0.713	0.455	0.399	0.146
276	V	0.456	0.544	0.547	0.074	0.379
277	S	0.531	0.469	0.562	0.39	0.048
278	F	0.818	0.182	0.739	0.023	0.238
279	D	0.85	0.15	0.823	0.151	0.026
280	A	0.849	0.151	0.875	0.023	0.101
281	D	0.7	0.3	0.827	0.1	0.074
282	T	0.338	0.662	0.541	0.246	0.213
283	I	0.113	0.887	0.291	0.236	0.473
284	R	0.06	0.94	0.202	0.595	0.203
285	I	0.053	0.947	0.289	0.224	0.486
286	A	0.113	0.887	0.446	0.387	0.167
287	Q	0.373	0.627	0.666	0.21	0.124

288	P	0.63	0.37	0.832	0.067	0.101
289	K	0.796	0.204	0.834	0.099	0.068
290	L	0.813	0.187	0.909	0.042	0.049
291	A	0.728	0.272	0.805	0.097	0.098
292	E	0.526	0.474	0.647	0.279	0.074
293	A	0.431	0.569	0.667	0.069	0.264
294	I	0.32	0.68	0.477	0.474	0.049
295	L	0.383	0.617	0.511	0.037	0.452
296	D	0.556	0.444	0.612	0.363	0.025
297	V	0.424	0.576	0.56	0.042	0.398
298	T	0.419	0.581	0.572	0.369	0.058
299	T	0.653	0.347	0.798	0.062	0.14
300	L	0.755	0.245	0.872	0.066	0.063
301	N	0.917	0.083	0.957	0.025	0.018
302	P	0.811	0.189	0.923	0.02	0.057
303	T	0.525	0.475	0.775	0.151	0.074
304	I	0.406	0.594	0.746	0.079	0.175
305	A	0.433	0.567	0.79	0.151	0.059
306	G	0.634	0.366	0.884	0.038	0.078
307	K	0.513	0.487	0.798	0.116	0.086
308	G	0.603	0.397	0.813	0.091	0.096
309	S	0.447	0.553	0.657	0.198	0.145
310	V	0.492	0.508	0.677	0.127	0.196
311	V	0.535	0.465	0.705	0.165	0.13
312	S	0.731	0.269	0.813	0.076	0.11
313	S	0.8	0.2	0.854	0.079	0.068
314	G	0.842	0.158	0.856	0.047	0.098
315	T	0.777	0.223	0.794	0.128	0.078
316	D	0.763	0.237	0.77	0.092	0.138
317	N	0.748	0.252	0.725	0.144	0.131
318	E	0.477	0.523	0.583	0.16	0.257
319	L	0.371	0.629	0.491	0.299	0.21
320	A	0.375	0.625	0.478	0.189	0.333
321	D	0.268	0.732	0.374	0.353	0.273
322	T	0.096	0.904	0.174	0.337	0.489
323	M	0.042	0.958	0.049	0.442	0.51
324	Q	0.018	0.982	0.027	0.574	0.399
325	I	0.009	0.991	0.012	0.454	0.534
326	V	0.008	0.992	0.016	0.475	0.509
327	S	0.011	0.989	0.07	0.618	0.312
328	L	0.01	0.99	0.058	0.259	0.682
329	Q	0.01	0.99	0.077	0.638	0.285
330	L	0.021	0.979	0.113	0.201	0.686
331	N	0.074	0.926	0.288	0.459	0.253
332	K	0.074	0.926	0.381	0.165	0.454
333	M	0.074	0.926	0.305	0.257	0.438
334	K	0.068	0.932	0.315	0.426	0.259
335	S	0.153	0.847	0.4	0.18	0.42
336	R	0.266	0.734	0.48	0.404	0.116

337	K	0.298	0.702	0.421	0.081	0.497
338	S	0.394	0.606	0.492	0.454	0.054
339	C	0.379	0.621	0.423	0.05	0.527
340	G	0.442	0.558	0.472	0.483	0.045
341	I	0.093	0.907	0.131	0.051	0.818
342	A	0.068	0.932	0.068	0.902	0.03
343	V	0.075	0.925	0.067	0.042	0.89
344	G	0.159	0.841	0.168	0.795	0.037
345	T	0.065	0.935	0.07	0.061	0.869
346	T	0.036	0.964	0.056	0.887	0.057
347	I	0.067	0.933	0.138	0.077	0.785
348	V	0.141	0.859	0.399	0.501	0.099
349	D	0.295	0.705	0.644	0.055	0.302
350	A	0.533	0.467	0.784	0.165	0.051
351	D	0.586	0.414	0.759	0.065	0.176
352	K	0.607	0.393	0.723	0.217	0.06
353	Y	0.39	0.61	0.362	0.054	0.584
354	A	0.334	0.666	0.188	0.788	0.024
355	V	0.285	0.715	0.102	0.02	0.878
356	T	0.22	0.78	0.068	0.918	0.014
357	V	0.317	0.683	0.123	0.013	0.864
358	E	0.223	0.777	0.114	0.874	0.012
359	A	0.099	0.901	0.065	0.015	0.92
360	R	0.106	0.894	0.104	0.877	0.019
361	L	0.072	0.928	0.139	0.022	0.839
362	I	0.113	0.887	0.29	0.66	0.051
363	D	0.321	0.679	0.6	0.025	0.375
364	E	0.42	0.58	0.676	0.292	0.032
365	R	0.467	0.533	0.562	0.045	0.393
366	A	0.37	0.63	0.357	0.597	0.046
367	A	0.272	0.728	0.195	0.035	0.77
368	H	0.211	0.789	0.103	0.857	0.041
369	V	0.22	0.78	0.058	0.019	0.922
370	N	0.388	0.612	0.072	0.913	0.015
371	A	0.297	0.703	0.067	0.015	0.917
372	Q	0.199	0.801	0.034	0.953	0.013
373	F	0.383	0.617	0.131	0.026	0.843
374	R	0.553	0.447	0.142	0.826	0.032
375	F	0.646	0.354	0.584	0.058	0.358
